# A Preliminary Evaluation of the Unified Protocol among Trauma-Exposed Adults with and without PTSD

**DOI:** 10.3390/ijerph182111729

**Published:** 2021-11-08

**Authors:** Caitlyn O. Hood, Matthew W. Southward, Christian Bugher, Shannon Sauer-Zavala

**Affiliations:** Department of Psychology, University of Kentucky, Lexington, KY 40506, USA; southward@uky.edu (M.W.S.); christian.bugher@uky.edu (C.B.); ssz@uky.edu (S.S.-Z.)

**Keywords:** posttraumatic stress disorder, trauma, transdiagnostic, cognitive-behavioral therapy

## Abstract

The purpose of this study was to evaluate whether the Unified Protocol (UP)—a mechanistically transdiagnostic psychological treatment—provides benefit to individuals with a range of trauma histories, psychological difficulties, and diagnostic comorbidity. Using data from a sequential multiple-assignment randomized trial (SMART), this exploratory analysis included a sample of 69 community-recruited adults seeking outpatient mental health treatment. We examined reductions in anxiety and depressive symptoms and changes in aversive and avoidant reactions to intense emotions—the UP’s putative mechanism—first by comparing individuals with and without trauma histories and then specifically among participants with PTSD. Findings suggest that the UP may lead to similar improvements in clinical diagnostic severity, anxiety, and depression among patients with trauma exposure as those without trauma exposure. Roughly half of participants with PTSD demonstrated reductions in PTSD clinical severity, anxiety, depression, and distress aversion, suggesting the UP may be an efficacious treatment for people with PTSD and comorbid conditions.

## 1. Introduction

Most adults will experience at least one potentially traumatic event involving exposure to actual or threatened death, serious injury, or sexual violence in their lifetime [1,2]. Although many individuals recover from the psychological effects of trauma within weeks or months, some experience debilitating and prolonged distress [3]. Posttraumatic stress disorder (PTSD) occurs when an individual exhibits persistent re-experiencing symptoms, efforts to avoid trauma reminders, negative changes in mood and cognition, and heightened arousal and reactivity [1]. However, trauma exposure is also associated with a range of mood and anxiety disorders beyond PTSD [4]. Diagnostic comorbidity is the rule, rather than the exception, among individuals with trauma histories and is associated with high levels of functional impairment and poor quality of life [5,6]. 

Evidence-based trauma-focused protocols (e.g., Cognitive Processing Therapy, Prolonged Exposure) are consistently recommended as the “gold standard” treatment approaches for PTSD [7]. Yet, there are several reasons why these protocols may not adequately address the needs of trauma-exposed individuals. First, some studies suggest that the efficacy of PTSD treatments may be compromised when diagnostic comorbidities or persistent and intense negative emotions are present. For example, treatment response may be hampered among individuals with primary diagnoses of PTSD and secondary diagnoses of major depressive or generalized anxiety disorder [8,9], as well as emotions like guilt, shame, or anger [9,10,11]. However, it should be noted that other studies suggest mood and anxiety comorbidities do not moderate outcomes for trauma-focused treatments and may even improve along with PTSD symptoms [7]. Second, evidence-based PTSD treatments are rarely available to individuals seeking psychotherapy in community settings [12]. Frontline clinicians may not have the time or resources to participate in the specialized training required for certification in evidence-based PTSD treatments or feel the need to provide such treatments if few patients on their caseloads report trauma-related symptoms. Third, patients may not want to undergo PTSD treatments that focus explicitly on the traumatic event or its consequences [13]. Thus, there is a need for alternative treatment approaches that can be easily embedded within community clinics and address the needs of individuals with an array of trauma-related psychopathology. 

Transdiagnostic treatments specifically target shared psychological processes that give rise to comorbid or co-occurring disorders [14,15]. One such treatment is the Unified Protocol for the Transdiagnostic Treatment of Emotional Disorders (UP) [16]. The UP includes five core skill modules that address aversive and avoidant reactions to intense emotions, which are thought to maintain symptoms across emotional disorders [17]. The UP is associated with large improvements in anxiety and depressive symptoms [18] comparable to single-disorder protocols [19]. Moreover, the UP can be adapted to meet the needs of various patient populations and clinical contexts [20]. Mechanistically transdiagnostic treatments, like the UP, may provide a parsimonious and efficient way of treating psychological difficulties among patients with complex comorbidity [21]. 

There is a strong theoretical and practical rationale for using mechanistically transdiagnostic treatments, like the UP, with trauma-exposed individuals [22,23,24]. Ample research points to emotion regulation and tolerance as factors implicated in the onset and maintenance of trauma-related psychopathology [25,26]. For example, individuals with poorer perceived ability to regulate or tolerate intense emotions—and their corresponding physical sensations—may be more likely to avoid trauma-related reminders, thus interfering with the cue exposure necessary for normative recovery following traumatic events [25,26,27]. The UP provides skills to develop an approach-oriented and willing stance towards experiencing emotions [28], which may counter avoidance of emotional, behavioral, and physiological responses to trauma-related reminders and promote adaptive recovery. Moreover, the UP may provide an efficient and effective treatment alternative for patients who are unwilling or unable to undergo a trauma-focused protocol. 

To date, two studies have evaluated the use of the UP among trauma-exposed adults with PTSD. Varkovitzky and colleagues [29] conducted an observational pilot study examining a 16-week group-based version of the UP embedded within a specialty PTSD clinic at a Veterans Affairs hospital. PTSD and depressive symptoms significantly decreased, and emotion regulation improved among the 52 veterans with military-related trauma who completed both the pre- and post-treatment assessments. O’Donnell and colleagues [30] expanded on this research in a randomized clinical trial comparing 10–14 individual sessions of the UP to usual care among 43 adults with traumatic injuries recruited from a community hospital. Compared to patients who received usual care, individuals who received the UP showed significantly larger reductions in PTSD, anxiety, and depression at both the post-treatment and 6-month follow-up assessments. Both studies provide preliminary evidence that the UP is efficacious for individuals with PTSD. 

Despite recent advances in using the UP for patients with PTSD from military or traumatic injury exposure, a question remains as to whether the UP provides benefit to individuals with a range of trauma histories, psychological difficulties, and diagnostic comorbidity. Using data from a sequential multiple-assignment randomized trial (SMART) [31], this exploratory analysis expands upon prior research by including a sample of treatment-naive community-recruited adults seeking outpatient mental health treatment. We examined reductions in anxiety and depressive symptoms and changes in aversive and avoidant reactions to intense emotions—the UP’s putative mechanism—first by comparing individuals with and without trauma histories and then specifically among participants with PTSD. Explicit hypotheses were withheld for the present study.

## 2. Materials and Methods

### 2.1. Participants 

Treatment-seeking individuals were recruited from the community to participate in a SMART evaluating methods of personalizing the delivery of the UP. Individuals were eligible for the parent study if they met criteria for at least one of the following Diagnostic and Statistical Manual-5 (DSM-5) disorders [1]: PTSD, generalized anxiety disorder (GAD), obsessive-compulsive disorder (OCD), major depressive disorder (MDD), panic disorder (PD), persistent depressive disorder (PDD), or social anxiety disorder (SAD). Of the 165 individuals who completed a preliminary eligibility screen via phone, 72 were excluded from participation due to having diagnoses that required clinical prioritization (e.g., uncontrolled bipolar disorder within the past year [n = 6], substance use disorder within the last 3 months [n = 1], current psychotic features [n = 7]), receiving five or more sessions of CBT within the last five years (n = 9), recent discontinuation of psychotropic medication (n = 1), being unwilling to pause current psychotherapy (n = 4), declining to participate (n = 33), living out of state (n = 2), or not meeting inclusion criteria (n = 9). Of the 93 individuals who completed the baseline assessment, 21 did not meet inclusion criteria and two declined to participate.

Seventy participants were eligible for the parent study at the baseline assessment and agreed to participate. Reports of trauma exposure were not available for one participant due to a recording malfunction, so the final sample in the present analyses included 69 participants. Demographics and clinical characteristics are shown in Table 1 and Table 2. Most of the sample was female, white, heterosexual, college-educated, and single. One-quarter of participants were on psychotropic medications. The most common principal diagnoses (i.e., rated as most distressing/interfering) were GAD, MDD, and SAD. Nine participants met diagnostic criteria for PTSD, and three had a principal diagnosis of PTSD. On average, participants met criteria for three concurrent diagnoses at baseline.

### 2.2. Procedure 

Informed consent was obtained before research activities began. All study procedures were approved by the University of Kentucky Institutional Review Board (Protocol code: 53545). The pilot parent study (NCT04584879) used a two-stage SMART design to address two primary aims: (1) examining the feasibility of personalizing UP skill module order and (2) identifying proximal indicators of early treatment response. The present study included the five core UP modules: Understanding Emotions (UE), Mindful Emotion Awareness (MEA), Cognitive Flexibility (CF), Countering Emotional Behaviors (CEB), and Confronting Physical Sensations (CPS). Weekly 45–60 min individual therapy sessions were delivered by four study therapists certified in the UP. 

The first-stage randomization occurred following the baseline assessment in which participants were randomized to receive the UP skill modules in one of three sequencing conditions: (1) a sequence that prioritized their personal strengths (capitalization condition); (2) a sequence that prioritized their personal deficits (compensation condition); or (3) the standard module sequence described by Barlow et al. [16] (standard condition). To sequence models in the capitalization and compensation conditions, self-report measures corresponding to each UP skill were administered as part of the baseline assessment battery (see summary of measures provided by Sauer-Zavala and colleagues [31]). Each participant’s raw scores on each measure were converted to z-scores and then rank-ordered from highest (greatest strength) to lowest (greatest deficit). The second-stage randomization occurred after the fifth therapy session, and patients were assigned to discontinue treatment after the sixth session (brief condition) or to receive all 12 sessions (full condition). 

Participants completed diagnostic assessments at three time points: (1) at baseline, (2) prior to the second-stage randomization (i.e., before session 6), and (3) at the end of the 12-week treatment window. Moreover, all participants completed a self-report questionnaire battery before each therapy session and, for those in the brief condition, each week between weeks 7–12. Additional information on procedures relevant to the main outcomes from the parent project, as well as a CONSORT flow chart can be found in Sauer-Zavala et al. [31].

### 2.3. Measures 

#### 2.3.1. Diagnostic Assessment

The Diagnostic Interview for Anxiety, Mood, and Obsessive-Compulsive and Related Neuropsychiatric Disorders (DIAMOND) [32] is a semi-structured diagnostic interview for DSM-5 disorders. Trained graduate student assessors used the DIAMOND to determine dimensional clinical severity ratings (CSR) of subjective distress and/or impairment for each diagnostic category. CSR scores use a seven-point scale (1–7) with higher scores reflecting greater distress/impairment. CSR scores > 3 represent clinically significant distress/impairment and were used to define the presence of a given diagnosis. The DIAMOND was administered at baseline to determine whether study inclusion/exclusion were met and to record baseline clinical severity. The DIMOND was also administered prior to the second-stage randomization (before week 6) and at the end of the 12-week treatment window. Assessors demonstrated excellent reliability on categorical ratings of primary diagnoses (Krippendorff’s αs: 0.91–1.00; median = 1.00) and dimensional severity ratings (CSRs) of each disorder (Krippendorff’s αs: 0.83–1.00; median = 0.92).

The DIAMOND was also used to assess participants’ lifetime exposure to potentially traumatic events. Assessors asked participants if they experienced any of the following: war or combat; actual or threatened physical assault; actual or threatened sexual violence; being kidnapped or held hostage; terrorist attack; being tortured or a prisoner of war; natural or man-made disaster; serious motor vehicle accident; a sudden, terrible medical event; or other traumatic experience. If an event was endorsed, participants were asked to report if they experienced the event directly, witnessed or learned about the event, or received repeated or extreme details of the event, which satisfies the definition of a Criterion A trauma outlined in DSM-5 [1]. Only participants who endorsed Criterion A trauma exposure were asked diagnostic items to determine the presence or absence of acute stress disorder (ASD) or PTSD.

#### 2.3.2. Self-Report

The Overall Anxiety Severity and Impairment Scale (OASIS) [33] and Overall Depression Severity and Impairment Scale (ODSIS) [34] are both 5-item self-report questionnaires designed to measure anxiety and depression symptoms, respectively, over the prior week. Total scores on the OASIS and ODSIS range from 0–20, with higher scores reflecting greater symptom severity. The clinical cutoff score for each measure is 8. The OASIS and ODSIS have exhibited good internal consistency, test-retest reliability, and convergent and discriminant validity [33,34]. In the current sample, OASIS items demonstrated good internal consistency (McDonald’s ω = 0.84) and ODSIS items demonstrated excellent internal consistency (McDonald’s ω = 0.94) at baseline. 

The 13-item Distress Aversion subscale of the Multidimensional Experiential Avoidance Questionnaire (MEAQ-DA) was used to assess the extent to which participants viewed distress as unwelcome or intolerable [35]. Participants used a Likert-type scale from 1 (strongly disagree) to 6 (strongly agree), with higher scores reflecting a greater propensity to experience aversive reactions to emotions—the UP’s putative mechanism. The MEAQ-DA has demonstrated good internal consistency and strong convergent and discriminant validity [35] in prior research. MEAQ-DA items demonstrated good internal consistency in the present study at baseline (McDonald’s ω = 0.89).

### 2.4. Data Analytic Approach 

Means and standard deviations were used to summarize participants’ average scores on continuous measures. Percentages were calculated to demonstrate participants’ exposure to traumatic events. Chi-square tests were used to compare whether the proportion of participants exposed to traumatic events differed by treatment conditions (sequencing or duration).

We assessed whether changes in anxiety, depression, and experiential avoidance across the study period differed as a function of trauma exposure by developing two-level hierarchal linear models (HLMs). HLMs can account for data that are nested within individuals (i.e., multiple time points per individual) and individuals with varying numbers of observations. Predictors in the models included main effects of trauma exposure (not exposed = 0, exposed = 1) and time. For models of CSR, time was operationalized as assessment number (baseline = 1, before session 6 = 2, end-of-study period = 3). For models of OASIS, ODSIS, and MEAQ-DA, time was operationalized as session number (1–12). To examine whether the rate of change in outcomes of interest differed by trauma exposure status, an interaction term between trauma and time (assessment or session number) was included in all models. Dummy-coded indicator variables representing sequencing and treatment duration conditions, and their interaction with time, were included as covariates. Data from the post-treatment assessment and sessions 7–12 were excluded for participants in the brief duration condition since they were not receiving treatment at those time points. Including a random intercept led to non-convergence when modeling change in CSR scores and, thus, only random slopes were included. All other models included both random intercepts and random slopes. We used restricted maximum likelihood estimation and applied an unstructured covariance structure to the residuals because this structure led to the best model fit as determined by Akaike Information Criterion. We used an intent-to-treat sample by including all participants who completed a baseline assessment. Analyses were conducted using SPSS version 28 [36] using the MIXED command. Study data collection and management were performed via REDCap [37]. Significance was assessed at α = 0.05 and adjustments for multiple testing were not applied to the reported *p*-values. All presented parameter estimates are unstandardized. 

A power analysis for CSR scores examining interactions of between- and within-person variables in a repeated measures design assuming power = 0.80, n = 69, 2 groups, 3 measurements, r = 0.50 correlation among repeated measures, and ε = 1.0 suggested we had enough power to detect effects as small as f = 0.15 (i.e., small-to-medium sized effects corresponding to d = 0.30). A power analysis for OASIS, ODSIS, and MEAQ-DA scores examining interactions of between- and within-person variables in a repeated measures design assuming power = 0.80, n = 69, 2 groups, 12 measurements, r = 0.60 correlation among repeated measures, and ε = 1.0 suggested we had enough power to detect effects as small as f = 0.09 (i.e., small sized effects corresponding to d = 0.18).

We examined the extent to which participants diagnosed with PTSD experienced significant within-person reductions in anxiety, depression, and distress aversion by computing 95% confidence intervals (CI) around observed change scores from pre- to post-treatment to determine reliability of changes [38]. Jacobson and Truax’s [39] method was used to calculate standard error of the difference (S_diff_), which reflects the difference between scores that is expected by chance variation alone on a given measure. Standard deviations and internal consistency coefficients from the following psychometric studies were used to calculate the S_diff_: OASIS (*SD* = 3.05, Cronbach’s α = 0.80) [33], ODSIS (*SD* = 5.04, Cronbach’s α = 0.94) [34], and MEAQ-DA (*SD* = 12.63, Cronbach’s α = 0.85) [35]. The S_diff_ was then multiplied by 1.96 to create a 95% CI around each change score. If the S_diff_ 95% CI does not include zero, the observed change is considered reliable rather than due to chance. Negative change scores indicate decreases on a given measure, and positive change scores indicate increases.

## 3. Results

### 3.1. Trauma Exposure 

The majority of participants in the sample (72.4%; n = 50) reported a history of trauma exposure. There were similar proportions of participants with trauma exposure among those who completed treatment and those who did not complete treatment, χ^2^ = 1.26, *p* = 0.74. Similarly, the proportion of trauma-exposed participants did not significantly differ by module-sequencing condition, χ^2^ = 1.63, *p* = 0.44, or duration condition, χ^2^ = 0.66, *p* = 0.42. As shown in Table 1, there was a larger proportion of people who identified as heterosexual among those with trauma exposure than those without trauma exposure (*p* = 0.01). However, there were no other significant differences in demographic variables between groups. The most frequently reported traumatic events were serious motor vehicle accidents, sudden, terrible medical events, and actual or threatened physical assault or sexual violence (Table 3). Among participants with a lifetime history of traumatic experiences, the average number of traumatic events reported was 1.52 (SD = 0.68), and the maximum number of events was 4.

We examined the intraclass correlations (ICCs) of our measures of interest to determine the proportion of variability in each measure attributable to between-person differences and the proportion attributable to within-person fluctuations. As mentioned above, the ICC for the CSR was unable to be calculated since including a random intercept term led to model non-convergence. The ICC for the OASIS was 0.36, indicating that 36% of the variability in this measure was due to between-person differences and 64% was due to within-person fluctuations. By contrast, the ICCs for ODSIS (0.57) and MEAQ-DA (0.80) indicated the majority of variability in these measures was between-persons. 

Models were first run with time (i.e., assessment or session number) as the only predictor. Participants demonstrated significant decreases on average across the study period in CSR scores, B = −1.44, SE = 0.14, *p* < 0.001, 95% CI [−1.72, −1.16], OASIS scores, B = −0.21, SE = 0.06, *p* = 0.002, 95% CI [−0.33, −0.08], ODSIS scores, B = −0.22, SE = 0.07, *p* = 0.002, 95% CI [−0.35, −0.09], and MEAQ-DA scores, B = −1.04, SE = 0.18, *p* < 0.001, 95% CI [−1.39, −0.69]. After adding all other relevant predictors, neither treatment order nor duration were significantly associated with changes in CSR, OASIS, ODSIS, or MEAQ-DA scores (*p*s > 0.05), and so the interactions between condition and time were not included in the models. Final models included time, trauma exposure, a time by trauma exposure interaction term, sequencing condition, and duration condition as predictors. Compared to participants without trauma histories, those exposed to potentially traumatic events reported significantly higher baseline CSR, B = 0.68, SE = 0.27, *p* = 0.01, 95% CI [0.14, 1.22], OASIS scores, B = 2.20, SE = 0.84, *p* = 0.01, 95% CI [0.51, 3.88], and ODSIS scores, B = 2.74, SE = 1.14, *p* = 0.02, 95% CI [0.45, 5.02]. However, trauma exposure was not significantly related to MEAQ-DA scores at baseline, B = −1.48, SE = 3.38, *p* = 0.66, 95% CI [−8.24, 5.27]. The interactions between trauma and time were also not significant, suggesting participants’ rate of change in CSR, B = −0.14, SE = 0.32, *p* = 0.67, 95% CI [−0.77, 0.50], OASIS scores, B = −0.11, SE = 0.14, *p* = 0.44, 95% CI [−0.38, 0.17], ODSIS scores, B = −0.28, SE = 0.14, *p* = 0.054, 95% CI [−0.57, 0.005], and MEAQ-DA scores, B = 0.26, SE = 0.39, *p* = 0.51, 95% CI [−0.53, 1.05], did not differ in magnitude between those with and without trauma exposure.

### 3.2. Posttraumatic Stress Disorder

Nine participants received diagnoses of PTSD (>3 CSR) at baseline (Table 4). Three participants had principal diagnoses of PTSD and the remaining six participants had principal diagnoses of GAD, MDD, OCD, PD, or SAD. On average, participants with PTSD met criteria for 4.89 total diagnoses (SD = 2.71). Seven participants diagnosed with PTSD reported experiencing physical (n = 6) or sexual assault (n = 1). One participant reported both physical and sexual assault exposure and one reported neither. On average, participants with PTSD reported 2.22 (SD = 0.83) types of potentially traumatic experiences. All patients with PTSD scored a 2 or above on CSR for GAD and/or MDD.

Outcome data at the post-treatment assessment were not available for one participant. Overall, the UP led to improvements for most participants; six of eight participants no longer met diagnostic criteria for PTSD at post-treatment (i.e., CSR scores < 3). On average, the UP led to decreases in anxiety (M_difference_ = −4.86, SD = 5.18), depression (M_difference_ = −4.14, SD = 4.45), and distress aversion (M_difference_ = −8.25, SD = 10.39) from pre- to post-treatment. Five participants demonstrated reliable reductions in anxiety and depression, and four participants demonstrated reliable reductions in distress aversion (Table 4).

## 4. Discussion

In this study, we examined whether the effects of a transdiagnostic treatment for emotional disorders (i.e., the UP) differed between people with and without a history of trauma exposure and whether this treatment would lead to significant improvements in diagnostic, symptomatic, and mechanistic outcomes among those with PTSD. Despite people with a history of trauma exposure reporting greater diagnostic clinical severity, anxiety, and depression at baseline than those without such a history, we found no significant differences in the rates of change on any outcome between groups. Further, the UP led to significant decreases in anxiety, depression, and distress aversion for roughly half of participants with PTSD.

In line with previous research [40], we found that participants with a history of trauma exposure demonstrated more severe clinical presentations and reported more severe anxiety and depression than participants without such a history at baseline. By contrast, these groups did not significantly differ in their ratings of distress aversion [41]. Together, these findings suggest that exposure to traumatic events may promote the development of more severe clinical symptoms, but other factors (e.g., maladaptive self-appraisals [42]; type of traumatic event [43]) may influence whether people develop or maintain greater aversions to distress after trauma exposure. Given that these results are cross-sectional, based on retrospective self-reported trauma exposure, and drawn from a treatment-seeking sample, we encourage future researchers to replicate these findings to determine their generalizability.

Trauma exposure did not significantly moderate the rate of change in diagnostic, symptom, or mechanistic outcomes. At the same time, the size of these differences varied substantially. Given that the beta weight of the interaction of time and trauma exposure represents the average difference in slopes between groups and the beta weight of time represents the average slope among the non-trauma exposed group, comparing the size of these beta weights can provide an indication of how different the slopes are. CSR slopes only differed by 11% between groups, whereas OASIS slopes differed by 52%, and ODSIS slopes differed by 127%. The direction of each interaction indicated that participants with trauma exposure experienced steeper rates of change on all three measures. By contrast, participants without trauma exposure experienced a 25% steeper rate of change in distress aversion. Together, these results indicate that the UP leads to similar diagnostic, clinical, and mechanistic improvements for patients regardless of trauma exposure, with some preliminary evidence of greater improvement in depression and anxiety but less improvement in distress aversion for patients with a trauma history. Our findings may imply that the UP is similarly efficacious at reducing clinical symptoms for people with and without trauma exposure but may act on a mechanism other than distress aversion to do so. Alternatively, given that clinical symptoms, but not distress aversion, were higher at baseline in the trauma-exposed group, these results may be due in part to regression to the mean. It is also possible that distress aversion is more resistant to change during treatment than clinical symptoms. We encourage future researchers to test these hypotheses more specifically by evaluating session-to-session changes in distress aversion as a potential mediator of clinical outcomes among a larger sample of people with and without trauma exposure.

Finally, this study provides preliminary support for continued investigation of the UP as a treatment for PTSD and comorbid conditions among trauma-exposed adults. Seven out of eight participants with PTSD at baseline demonstrated decreases in the severity of their PTSD diagnoses across treatment. Five participants demonstrated significant reductions in anxiety, and six participants reported OASIS scores below the suggested clinical threshold. Similarly, five participants demonstrated significant reductions in depression, and seven participants reported ODSIS scores below the suggested clinical threshold. Finally, four participants demonstrated significant reductions in distress aversion, three of whom also reported significant reductions in anxiety and depression. These results provide preliminary evidence for the generalizability of the UP for PTSD beyond those with military or traumatic injury exposure. Our results further suggest the UP may be efficacious for comorbid anxiety and depression among those presenting with primary PTSD, in line with the transdiagnostic nature of this treatment protocol. To support this claim, we encourage researchers to test the non-inferiority of the UP for PTSD compared to “gold standard” trauma-focused treatments—similar to Barlow and colleagues’ [19] non-inferiority test of the UP for anxiety disorders compared to single-disorder anxiety treatment protocols—in a fully-powered randomized clinical trial among individuals with PTSD diagnoses from various traumatic event exposure. The UP may indeed work well to reduce clinical symptoms among trauma-exposed adults without PTSD, whereas other treatment approaches may be more appropriate when PTSD is present. Future research should be designed to test this question by comparing the effectiveness of the UP among individuals without trauma histories to trauma-exposed individuals with and without PTSD.

These results should be considered in light of the study’s limitations. First, our only direct measures of PTSD were clinician-reported diagnostic and severity ratings. Although we assessed PTSD criteria and severity at pre-, mid-, and post-treatment among those diagnosed with PTSD at baseline, we encourage future researchers to improve upon these assessments by using “gold-standard” diagnostic (e.g., the Clinician-Administered PTSD Scale [44]) and more frequent self-report symptom assessments (e.g., the PTSD Checklist for DSM-5 [45]) to specifically test how putative mechanisms in the UP directly impact changes in PTSD symptomology. Second, trauma exposure was assessed retrospectively and at the same time as diagnostic, clinical, and mechanistic outcomes, and so we cannot determine the temporal precedence of these factors. Because we did not systematically assess when trauma exposure occurred, we are unable to determine whether the chronicity of trauma exposure is associated with greater comorbidity or differences in treatment response. Third, our ability to generalize findings to the broader population is limited due to the parent project’s exclusion criteria, the small number of participants with PTSD, and the predominantly white and college-educated sample.

## 5. Conclusions

Our findings add to the burgeoning literature on the use of the UP as a possible treatment for PTSD and comorbid conditions among trauma-exposed adults. Results suggest the UP may lead to similar, if not larger, improvements in clinical severity, anxiety, and depression among patients with trauma exposure compared to those without trauma exposure. We found our results despite patients with trauma histories reporting higher levels of these characteristics at baseline. There were no significant differences between groups in the rate of improvement in distress aversion, suggesting that this mechanism may also respond similarly to the UP among people with and without trauma exposure. Finally, we found that roughly half of participants with PTSD demonstrated reductions in PTSD clinical severity, anxiety, depression, and distress aversion, highlighting the potential of the UP as an efficacious treatment for people with PTSD and comorbid conditions.

## Figures and Tables

**Table 1:** Baseline Demographic Characteristics.

Characteristic	Total (N = 69)	Trauma History (n = 50)	No Trauma History (n = 19)	PTSD Diagnosis (n = 9)	No PTSD Diagnosis (n = 60)
Age (*M, SD*)	33.46 (12.51)	35.20 (12.45)	28.89 (11.78)	36.56 (12.19)	33.00 (12.59)
Gender					
Female	46 (66.7)	34 (68.0)	12 (63.2)	5 (55.6)	41 (68.3)
Male	22 (31.9)	16 (32.0)	6 (31.6)	4 (44.4)	18 (30.0)
Genderqueer/Non-binary	1 (1.4)	0 (0.0)	1 (5.3)	0 (0.0)	1 (1.7)
Racial/Ethnic Background ^a^					
Caucasian	51 (73.9)	36 (72.0)	15 (78.9)	8 (88.9)	43 (71.7)
African-American	9 (13.0)	8 (16.0)	1 (5.3)	0 (0.0)	9 (15.0)
Arab/Middle-Eastern American	2 (2.9)	2 (4.0)	0 (0.0)	0 (0.0)	2 (3.3)
East Asian	3 (4.3)	2 (4.0)	1 (5.3)	0 (0.0)	3 (5.0)
Latinx	2 (2.9)	1 (2.0)	1 (5.3)	1 (11.1)	1 (1.7)
South Asian	2 (2.9)	1 (2.0)	1 (5.3)	0 (0.0)	2 (3.3)
Heterosexual/Straight	51 (73.9)	41 (82.0) *	10 (52.6)	6 (66.7)	45 (75.0)
Bachelor’s Degree or Higher	41 (59.3)	32 (64.0)	9 (47.4)	5 (55.6)	36 (60.0)
Married	22 (31.9)	19 (38.0)	3 (15.8)	3 (33.3)	19 (31.7)

Note. Data are presented as number (percentage) of patients unless otherwise indicated. ^a^ Values may not sum to total in each column because participants could select multiple racial/ethnic backgrounds. * *p* < 0.05.

**Table 2:** Baseline Diagnostic Characteristics.

Characteristic	Total (N = 69)	Trauma History (n = 50)	No Trauma History (n = 19)	PTSD Diagnosis (n = 9)	No PTSD Diagnosis (n = 60)
Current Psychotropic Medication	16 (23.2)	11 (22.9)	5 (29.4)	4 (44.4)	12 (21.4)
Principal Diagnoses ^a^					
Obsessive-Compulsive Disorder	5 (7.2)	5 (10.0)	0 (0.0)	1 (11.1)	4 (6.7)
Social Anxiety Disorder	16 (23.2)	12 (24.0)	4 (21.1)	3 (33.3)	13 (21.7)
Generalized Anxiety Disorder	32 (46.4)	24 (48.0)	8 (42.1)	2 (22.2)	30 (50.0)
Panic Disorder	4 (5.8)	4 (8.0)	0 (0.0)	1 (11.1)	3 (5.0)
Agoraphobia	1 (1.4)	1 (2.0)	0 (0.0)	0 (0.0)	1 (1.7)
Major Depressive Disorder	19 (27.5)	13 (26.0)	6 (31.6)	3 (33.3)	16 (26.7)
Persistent Depressive Disorder	12 (17.4)	9 (18.0)	3 (15.8)	2 (22.2)	10 (16.7)
Acute Stress Disorder ^b^	1 (1.4)	1 (2.0)	-	-	1 (1.7)
Posttraumatic Stress Disorder ^b^	3 (4.3)	3 (6.0)	-	3 (33.3)	-
Diagnoses Met (*M*, *SD*)	3.04 (1.80)	3.28 (1.97) *	2.42 (1.07)	2.77 (1.47)	4.89 (2.71) *
Clinical Severity Rating (*M*, *SD*)	4.72 (1.00)	4.92 (0.94) **	4.21 (0.98)	4.65 (0.95)	5.22 (1.20)
OASIS (*M*, *SD*)	9.18 (3.68)	9.84 (3.59) *	7.47 (3.42)	8.97 (3.44)	10.75 (5.15)
ODSIS (*M*, *SD*)	8.28 (5.09)	8.80 (5.10)	6.95 (4.95)	8.02 (5.06)	10.25 (5.18)
MEAQ-DA *(M, SD)*	47.26 (11.92)	47.32 (12.70)	47.10 (9.88)	47.18 (12.37)	47.78 (8.84)

Note. Data are presented as number (percentage) of patients unless otherwise indicated. ^a^ Values may not sum to total in each column because participants could be diagnosed with multiple clinically significant diagnoses. ^b^ Participants were only asked diagnostic items for ASD and PTSD if they reported a Criterion A trauma. ASD and PTSD are mutually exclusive diagnoses. OASIS = Overall Anxiety Severity and Impairment Scale. ODSIS = Overall Depression Severity and Impairment Scale. MEAQ-DA = Multidimensional Experiential Avoidance Questionnaire Distress Aversion subscale. * *p* < 0.05 ** *p* < 0.01.

**Table 3:** Trauma Characteristics.

Type of Traumatic Event	Type of Trauma Exposure						Total N = 69	
	Experienced		Witnessed Directly or Learned about		Received Repeated or Extreme Details			
	n	%	n	%	n	%	n	%
Exposure to war or combat	0	0	1	1.4	0	0	1	1.4
Actual or threatened physical assault	9	13.0	11	15.9	0	0	18	26.1
Actual or threatened sexual violence	9	13.0	5	7.2	0	0	14	20.3
Being kidnapped or held hostage	0	0	1	1.4	0	0	1	1.4
Terrorist attack	0	0	0	0	0	0	0	0.0
Being tortured or a prisoner of war	0	0	0	0	0	0	0	0.0
Natural or man-made disaster	1	1.4	0	0	0	0	1	1.4
Serious motor vehicle accident	12	17.4	10	14.5	0	0	20	29.0
A sudden, terrible medical event	6	8.7	15	21.7	0	0	18	26.1
Other traumatic event	0	0	1	1.4	2	2.9	3	4.3

Note. Values may not sum to total in each row because participants could report multiple types of exposure to the same traumatic event.

**Table 4:** Change in Anxiety, Depression, and Distress Aversion Among Participants with PTSD Diagnoses.

Case	PTSD CSR		OASIS 95% CI = *CS* ± 3.78			ODSIS 95% CI = *CS* ± 3.42			MEAQ-DA 95% CI = *CS* ± 13.78		
	Pre	Post	Pre	Post	Change Score	Pre	Post	Change Score	Pre	Post	Change Score
Case 1 ^s,b^	5	-	18	-	-	19	-	-	53	-	-
Case 2 ^ca,f^	4	5	9	10	1 (−2.78, 4.78)	8	10	2 (−1.42, 5.42)	53	53	0 (−13.78, 13.78)
Case 3 ^†,ca,b^	6	2	11	1	−10 * (−13.78, −6.22)	5	0	−5 * (−8.42, −1.58)	38	46	8 (−5.78, 21.78)
Case 4 ^†,s,f^	4	2	3	3	0 (−3.78, 3.78)	5	4	−1 (−4.42, 2.42)	33	19	−14 * (−27.78, −0.22)
Case 5 ^co,f^	6	2	18	5	−13 * (−16.78, −9.22)	17	5	−12 * (−15.42, −8.58)	48	34	−14 * (−27.78, −0.22)
Case 6 ^†,co,f^	4	3	7	5	−2 (−5.78, 1.78)	8	6	−2 (−1.42, 5.42)	51	46	−5 (−18.78, 8.78)
Case 7 ^s,b^	3	1	11	5	−6 * (−9.78, −2.22)	10	5	−5 * (−8.42, −1.58)	44	27	−17 * (−30.78, −3.22)
Case 8 ^co,b^	3	2	9	5	−4 * (−7.78, −0.22)	10	4	−6 * (−9.42, −2.58)	47	46	−1 (−14.78, 12.78)
Case 9 ^s,f^	4	2	12 ^‡^	8	−4 * (−7.78, −0.22)	12 ^‡^	7	−5 * (−8.42, −1.58)	63	40	−23 * (−36.78, −9.22)

Note. Negative change scores indicate decreases on a given measure, and positive change scores indicate increases. The standard error of the difference is listed at the top of each column and reflects the value used to calculate the 95% CIs around each raw change score. CI = confidence interval; CS = change score; CSR = Clinical Severity Rating; OASIS = Overall Anxiety Severity and Interference Scale; ODSIS = Overall Depression Severity and Interference Scale; MEAQ-DA = Multidimensional Experiential Avoidance Questionnaire Distress Aversion subscale. ^b^ Brief duration condition. ^ca^ Capitalization sequencing condition. ^co^ Compensation sequencing condition. ^f^ Full duration condition. ^s^ Standard sequencing condition. ^†^ Participant had a principal diagnosis of PTSD. ^‡^ Participant did not complete the OASIS and ODSIS at the first assessment, so data collected at baseline were substituted. * Indicates statistically significant decrease.

## Data Availability

Raw data are available upon request. Syntax files for the primary outcomes are located on Open Science Framework (https://osf.io/xrv8k/) (accessed on 8 November 2021).
